# Alternative Activation of Macrophages and Induction of Arginase Are Not Components of Pathogenesis Mediated by Francisella Species

**DOI:** 10.1371/journal.pone.0082096

**Published:** 2013-12-06

**Authors:** Amanda J. Griffin, Deborah D. Crane, Tara D. Wehrly, Dana P. Scott, Catharine M. Bosio

**Affiliations:** 1 Immunity to Pulmonary Pathogens Section, Laboratory of Intracellular Parasites, Rocky Mountain Veterinary Branch, Rocky Mountain Laboratories, National Institute of Allergy and Infectious Diseases, National Institutes of Health, Hamilton, Montana, United States of America; 2 Veterinary Pathology Section, Rocky Mountain Veterinary Branch, Rocky Mountain Laboratories, National Institute of Allergy and Infectious Diseases, National Institutes of Health, Hamilton, Montana, United States of America; Université Paris Descartes, INSERM, France

## Abstract

Virulent *Francisella tularensis* ssp *tularensis* is an intracellular, Gram negative bacterium that causes acute lethal disease following inhalation of fewer than 15 organisms. Pathogenicity of Francisella infections is tied to its unique ability to evade and suppress inflammatory responses in host cells. It has been proposed that induction of alternative activation of infected macrophages is a mechanism by which attenuated Francisella species modulate host responses. In this report we reveal that neither attenuated *F. tularensis* Live Vaccine Strain (LVS) nor virulent *F. tularensis* strain SchuS4 induce alternative activation of macrophages in vitro or in vivo. LVS, but not SchuS4, provoked production of arginase1 independent of alternative activation in vitro and in vivo. However, absence of arginase1 did not significantly impact intracellular replication of LVS or SchuS4. Together our data establish that neither induction of alternative activation nor expression of arginase1 are critical features of disease mediated by attenuated or virulent Francisella species.

## Introduction

*Franicsella tularensis* is a Gram-negative, facultative intracellular pathogen and the causative agent of tularemia. There are two clinically relevant subspecies that cause disease in humans. Subspecies *holartica* can infect humans, but does not typically result in lethal disease [1]. In contrast, infection with subspecies *tularensis* can be lethal following inhalation of fewer than 15 organisms [2]. Due to the high virulence of subspecies *tularensis*, its low infectious dose, and its ability to cause infection following aerosolization, it was developed as a biological weapon [3]. Tularemia can be treated with antibiotics. However, recrudescence of infection following cessation of antibiotic therapy is not uncommon [4]. Survival of tularemia does not result in complete immunity from subsequent infection and currently there are no licensed vaccines approved for human use [5]. Thus, there is still a need for novel therapeutics and vaccines to protect against tularemia.

As an intracellular pathogen, *F. tularensis* must infect and replicate in host cells. The primary target cells for *F. tularensis* include, but are not limited to, macrophages and dendritic cells [6]. An important mechanism of virulence for *F. tularensis* is its ability to evade, suppress, and modulate activation of these host cells [7]–[10]. It has been suggested that attenuated Francisella species directly trigger alternative activation pathways in host macrophages as a strategy to suppress protective inflammatory responses [11]. However, it is not known if virulent *F. tularensis* utilizes a similar tactic to inhibit inflammation.

Macrophages exhibit great plasticity in their activation state. Among these conditions two widely accepted and studied activation states are classical and alternative activation. Classical activation of macrophages occurs following exposure to Th1 type cytokines such as IFN-γ [12]. Macrophages activated in this manner effectively control and kill intracellular pathogens [12]. In contrast to Th1 driven classically activated macrophages, a strict definition of alternatively activated macrophages (AAMs) entails induction via by Th2 cytokines, e.g. IL-4 and IL-13, and increased expression of three specific genes encoding Arginase1 (Arg1), Ym-1 and FIZZ-1 [12]. AAMs down modulate expression of Th1 type cytokines and, thus, have been implicated in the exacerbation of bacterial infection that rely on classically activated macrophages for resolution of disease [12].

Recently, it was suggested that attenuated *F. tularensis* ssp *holartica* Live Vaccine Strain (LVS) directly induced alternative activation of macrophages via induction of IL-4 by infected macrophages as a strategy to cause lethal disease [11]. Although there are clear differences in the pathogenesis and ability of virulent *F. tularensis* to modulate inflammatory responses compared to attenuated LVS, control of intracellular replication of both bacteria is dependent on the Th1 cytokine IFN-γ [13], [14]. Therefore, it is possible that dysregulation of IFN-γ responsiveness among AAMs may be beneficial to virulent *F. tularensis* as is suggested for LVS.

In this report we directly compared the ability of LVS and virulent *F. tularensis* strain SchuS4 to induce alternative activation of primary macrophages in vitro and in the mouse lung in vivo. While there was no evidence that either strain successfully triggered AAMs in vitro or in vivo, LVS infection did promote induction of Arg1 in host cells. However, our data also show that Arg1 did not play a significant role in intracellular replication of either LVS or SchuS4. Thus, neither provocation of alternative activation nor independent induction of Arg1 are important features of pathogenesis mediated by various Francisella species.

## Materials and Methods

### Bacteria

*F. tularensis* subsp. *tularensis* strain SchuS4 was originally provided by Dr. Jeannine Peterson (Centers for Disease Control and Prevention, Fort Collins, CO). *F. tularensis* subsp. *holarctica* Live Vaccine Strain (LVS) American Type Culture Collection (ATCC) 29684, was provided by Dr. Karen Elkins (U.S. Food and Drug Administration, Rockville, MD). Bacterial stocks were generated as previously described [7]–[9]. Briefly, bacteria were grown overnight in modified Mueller-Hinton (MMH) broth, and 1-ml aliquots were frozen at −80°C. Bacteria were thawed just prior to use. As previously described, frozen stocks were titered by enumerating viable bacteria from serial dilutions plated on MMH agar [7]–[9]. The number of viable bacteria in frozen stock vials varied by <1% over a 10-month period.

### Ethics Statement

All research involving mice was conducted in accordance with Animal Care and Use guidelines and animal protocols were approved by the Animal Care and Use Committee at Rocky Mountain Laboratories.

### Mice

Specific-pathogen-free, 6–8 week old C57Bl/6J mice were purchased from Jackson Laboratories (Bar Harbor, ME). Mice were housed in sterile microisolator cages in the BSL-3 facility at the RML. All research involving mice was conducted in accordance with Animal Care and Use guidelines and animal protocols were approved by the Animal Care and Use Committee at RML. Infected mice were monitored regularly and euthanized at the first sign of illness as determined by the Animal Care and Use guidelines. Bones from Arg1^flox/flox^ (wild type controls for Arg1^-/-^ [15]) and Arg1^flox/flox^-Tie2Cre (Arg1^-/-^) mice were kindly provided by Dr. Peter Murray (St. Jude Childrens Research Hospital, Nashville, TN).

### Generation and infection of bone marrow-derived macrophages

Bone marrow-derived macrophages (BMDM) were generated as previously described [16]. Briefly, femurs from 6–8 week old C57BL/6J mice were flushed and red blood cells lysed. Cells were washed and suspended in complete Dulbecco's minimum essential medium (cDMEM, DMEM supplemented with 10% heat-inactivated fetal calf serum (FCS [Thermo]), 0.2 mM 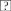-glutamine, 1 mM HEPES, and 0.1 mM non-essential amino acids (NEAA [Gibco]), and incubated in the presence of 20 ng/ml M-CSF (Peprotech) in a 75 cm^2^ flask for two days. Non-adherent cells were then centrifuged, resuspended in cDMEM plus 20 ng/ml M-CSF, and incubated for two days in a 75 cm^2^ flask. Media and cytokine was changed and the cells were again incubated overnight. Cells were gently scraped off of the flask, counted, and plated at 1×10^5^ cells/well in 48-well plates. Macrophages were infected as previously described, but with the following exceptions [17]. Briefly, the conditioned medium (cDMEM plus M-CSF) was gently removed from the wells and reserved. SchuS4 or LVS was diluted from frozen stocks in cDMEM and BMDM were infected at a multiplicity of infection (MOI) of 1, 10, or 50 Colony Forming Units (CFU) SchuS4 or LVS for 90 minutes at 37°C/7% CO_2_. The infection inoculum was confirmed by plating serial dilutions on MMH agar plates immediately following infection. BMDM were then incubated with 50 µg/ml gentamicin in cDMEM for 45 minutes to eliminate extracellular bacteria, washed three times with PBS, and reserved media was added to each well. Cells were incubated at 37°C/7% CO_2_ for the remainder of the experiment. Intracellular bacteria were enumerated by lysing cells at the indicated time point with sterile water followed by serial dilution of lysates and inoculation of dilutions onto MMH agar plates. Plates were incubated at 37°C/7% CO_2_ for 48 hours before individual colonies were counted. As indicated, uninfected BMDM were treated with 20 ng/ml recombinant murine IL-4 (Peprotech) prior to assessment for changes in expression of genes listed below.

### Fluorescence microscopy

Macrophages were grown on coverslips, treated, and infected with LVS or SchuS4 as described above. Cells were fixed in 3% paraformaldehyde for 30 minutes at 37°C/5%CO_2_. Cells were washed with PBS and stained for LAMP-1 as previously described [18], [19]. LVS and SchuS4 were detected using Alexa Fluor488 goat conjugated anti-*F. tularensis* (US Biological, Swampscot, MA) as previously described [18]. Samples were observed on a Carl Zeiss (Thornwood, NY) Axio Imager.M1 epifluoresence microscope for quantitative analysis. Approximately 75–100 cells/field and a minimum of three fields per coverslip were analyzed for presence of intracellular bacteria. Percent of infected cells was calculated as follows: (number infected cells/total number of cells) x 100.

### Quantification of transcripts by qRT-PCR

At the indicated time points, total RNA was extracted and purified from cell cultures or organ homogenates using an RNeasy Plus Mini Kit (Qiagen). cDNA was generated using a Superscript VILO cDNA Synthesis Kit (Invitrogen), according to the manufacturer's instructions. Real-time quantitative PCR was performed using primer/probe sets for mouse Arg1, FIZZ-1, Ym-1, and GAPDH (TaqMan Gene Expression Assays, Applied Biosystems). Input RNA was normalized to GAPDH, and fold change was quantified as ΔΔCT for infected cells normalized to mock-infected cells.

### Cytokine Quantification

Concentrations of IL-4, IL-10, IL-6, IL-12p40, IL-5 (BD Biosciences, San Jose, CA), and IL-13 (R&D Systems, Minneapolis, MN) in cell culture supernatants were determined by ELISA using commercially available kits following manufacturer's instructions. Absorbance at 450 nm was determined and concentrations quantified using a Dynex MRX Revelation instrument and Revelation software (Dynex Technologies, Chantilly, VA). Concentrations of IL-4, IL-5, and IL-13 in bronchoalveolar lavage fluid were assessed using cytometric bead array flex sets (BD Biosciences) according to manufacturer's instructions.

### Infection of mice

Mice were infected with the indicated strains of Francisella as previously described [8], [13]. Briefly, bacteria were thawed and serially diluted in PBS. Mice were anesthetized by intraperitoneal injection of 70 µl of 12.5 mg/ml ketamine + 3.8 mg/ml xylazine. Then, mice were immediately infected intranasally with approximately 40 CFU/25 µl *F. tularensis* SchuS4 (∼40 LD50s) or 2×10^6^ CFU/25 µl *F. tularensis* LVS strain ATCC 29684 (∼200 LD50s). Actual inoculum concentration was confirmed by plating an aliquot of the inoculum onto MMH agar plates, incubating the plates at 37°C/7%CO_2_ for 48 hours and enumerating colonies. As indicated, uninfected animals were given 2.5 µg/25 µl of schistosomal egg antigen (SEA) diluted in pharmaceutical grade saline (SEA; a kind gift from Dr. Tom Wynn) intranasally on days −7, −5, −3, and −1 prior to collection of tissues for assessment of eosinophilia and changes in expression of the indicated genes. All experiments using mice were performed in accordance with protocols approved by the Animal Care and Use Committee at RML under ABSL-3 conditions.

### Enumeration of bacteria in vivo

Bacteria were enumerated from the lungs as previously described [13], with the following modifications. Briefly, organs were aseptically collected and placed in ice cold PBS. Organs were homogenized by grinding tissues through a sterile S/S Type 304 #60 wire mesh screen (Billeville Wire Cloth Co., Cedar Grove, NJ) using a 3 ml syringe plunger. An aliquot of the resulting homogenate was serially diluted in PBS and plated on MMH agar for enumeration of bacterial loads.

### Assessment of pathological changes in tissues

Tissues were assessed for pathological changes as previously described [13]. Briefly, tissues were fixed in 10% neutral buffered formalin for a minimum of 24 hours. Tissues were then embedded in paraffin, placed in cassettes, and processed with a Sakura VIP-5 Tissue Tek, on a 12 hour automated schedule, using a graded series of ethanol, xylene, and ParaPlast Extra. Embedded tissues were sectioned at 5 µm and dried overnight at 42°C prior to staining. Tissue sections were stained with hematoxylin and eosin (H&E) and examined on an Olympus BX51 light-microscope equipped with an Olympus DP722 camera and associated cellSens Dimension 1.4.1 software. Tissues were assessed by a board certified pathologist for presence and extent of eosinophilia. Extent and distribution of eosinophilic lesions in each lung (n = 5/group) was scored on a scale of 1–10 to obtain a total score for each group of animals.

### Statistical Analysis

Statistical differences between two groups were determined using an unpaired two-tailed *t* test, with significance set at p<0.05.

## Results

### Absence of cytokines characteristic of AAMs following in vitro infection with LVS and SchuS4

**Figure 1:**
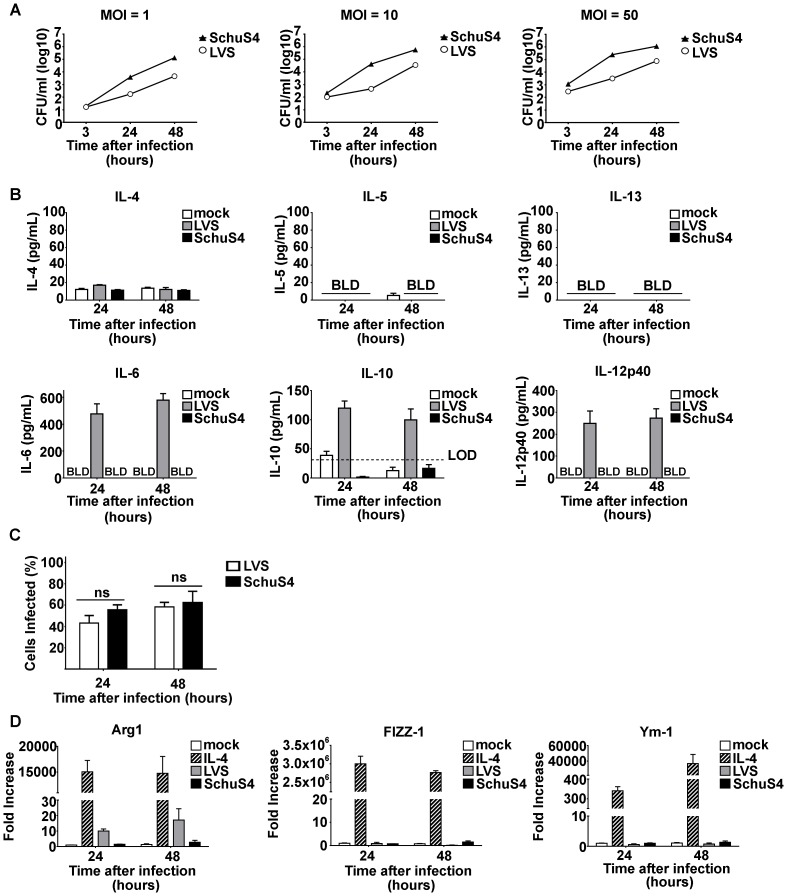
Francisella does not induce alternative activation of macrophages in vitro. (A) Primary BMM were infected with the indicated MOI of LVS or SchuS4 and intracellular replication was monitored over time. (B) Supernatants from cultures of BMM infected with LVS or SchuS4 at MOI  = 50 were assessed for the indicated cytokines 24 hours after infection. Mock infected cells served as negative controls. (C) Percent infected cells from culture infected with a MOI  = 50 were evaluated by microscopy. (D) Changes in expression of Arg1, Ym-1 and FIZZ-1 were analyzed from BMM infected with MOI = 50 of LVS or SchuS4 compared to mock infected controls. Each data point represents the mean of triplicate samples. *  =  significantly different from mock treated controls (p<0.05). Error bars represent SEM. LOD  =  limit of detection. BLD  =  below level of detection. Data is representative of three experiments of similar design.

Induction of AAMs is dependent on the presence of IL-4, IL-5, and IL-13. Thus, we first compared the ability of LVS and SchuS4 to induce secretion of these cytokines following infection of mouse and human cells. Primary macrophages were infected with various MOIs and secretion of cytokines into culture supernatant was assessed over time. As expected, both LVS and SchuS4 replicated in macrophages (Figure 1A). We did not detect cytokines in culture supernatants from BMM infected with MOI  = 1 or 10 (data not shown). Further, we did not detect the presence of IL-4, IL-5 or IL-13 in culture supernatants at any time after infection among cells infected with MOI  = 50 with LVS or SchuS4 (Figure 1B). LVS did provoke production of IL-10, IL-6 and IL-12p40 at MOI  = 50 demonstrating that the macrophages were capable of sensing LVS (Figure 1B). As previously demonstrated, SchuS4 evaded detection by macrophages and did not induce secretion of IL-10, IL-6 or IL-12p40 (Figure 1B and [20]). Similar percentages of BMDM were infected with LVS or SchuS4 (Figure 1C). Thus, differences in the ability of LVS versus SchuS4 to provoke cytokine production from BMDM were not a result of fewer infected cells present in the culture.

### Neither LVS nor SchuS4 induce genes characteristic of AMMs in vitro

Despite the absence of detectable IL-4, IL-5 or IL-13 in culture supernatants of Francisella infected cells, it was possible that concentrations of cytokines below the level of detection were present and capable of inducing AMMs. AAMs are characterized by the expression of Arg1, FIZZ-1 and Ym-1 [12]. Therefore, we next determined if Francisella infection resulted in increased expression of these genes. Cells were infected with multiple MOIs of LVS or SchuS4 and changes in expression of Arg1, FIZZ-1 and Ym-1 were monitored over time by qRT-PCR. As previously shown, macrophages treated with IL-4 induced an AAM program as evidenced by the significantly increased expression of Arg1, FIZZ-1 and Ym-1 compared to mock treated controls (Figure 1D). In contrast to IL-4 treated cells, we did not observe changes in gene expression among cells infected with MOI  = 1 or 10 (data not shown). Further, neither LVS nor SchuS4 induced FIZZ-1 or Ym-1 in macrophages following infection with MOI  = 50 (Figure 1D). LVS reproducibly induced increased expression of Arg1 among cells in vitro (Figure 1D). We also evaluated the percent of cells infected at 24 and 48 hours after infection to determine if lack of increased expression of FIZZ-1 and Ym-1 could be attributed to low numbers of infected cells. However, at a MOI = 50 approximately 50–60% of cells were infected with either LVS or SchuS4 24 and 48 hours after infection, respectively (Figure 1C). Thus, lack of detection of changes in expression of FIZZ-1 or Ym-1 was not due to low numbers of infected cells. Together, our data demonstrate that Francisella does not induce AAMs following in vitro infection. However, there are distinct differences in the ability of LVS and SchuS4 to provoke Arg1 independent of alternative activation in primary macrophages.

### Francisella does not induce alternative activation in vivo

**Figure 2:**
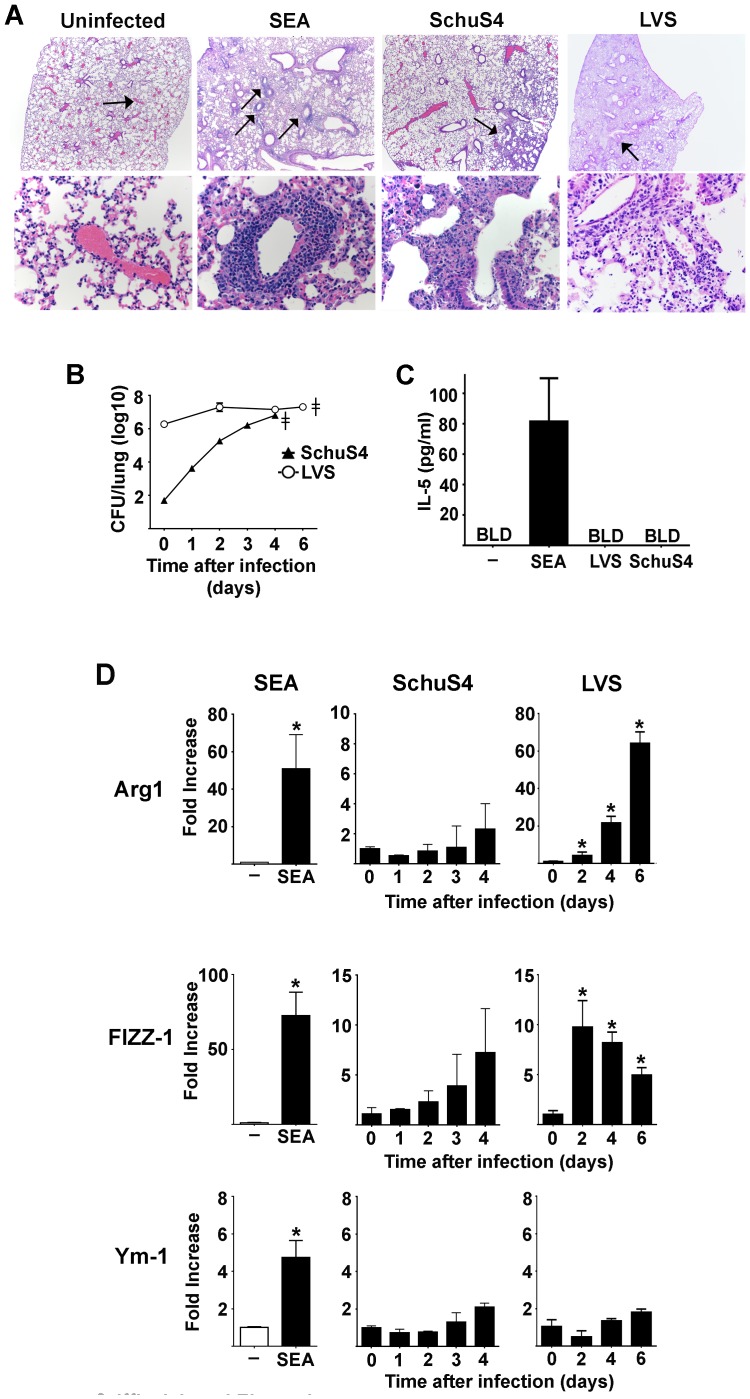
Francisella does not induce alternative activation of macrophages in vivo. Mice (n = 5/group) were intranasally infected with approximately 40 LD50s SchuS4 or 200 LD50s LVS. (A) Mice were euthanized on day 4 and 6 after infection, respectively. The lungs were collected and assessed for eosinophils via histopathological analysis. Mice treated with SEA served as positive controls (n = 5). The upper panel represents pictograms at 40× magnification. The lower panel represents the locations indicated by arrows in the 40× panels at 400× magnification. SEA treated mice show distinct perviascular infiltrates of eosinophils. No eosinophils were evident in lesions observed in LVS or SchuS4 infected animals. At the indicated time points after infection lungs were evaluated for CFU (B), bronchoalveolar lavage fluid was collected and assessed for IL-5 after infection (day 4 results depicted)or 24 hours after last administration of SEA (C), and lung tissue was analyzed for changes in expression of Arg1, Ym-1 and FIZZ-1 compared to mock treated controls (D). Mice treated with SEA served as positive controls (n = 5). *  =  significantly different from mock treated controls (p<0.05). ???  =  time at which mice showed signs of illness requiring euthanasia. BLD  =  below level of detection. Error bars represent SEM. Data is representative of two experiments of similar design.

As described above, macrophages represent an important primary cell type targeted by Francisella for in vivo infection, thus justifying use of these cells for in vitro study of Francisella-host cell interactions. However, the in vivo environment also consists of other cell types that may contribute to the overall activation state of macrophages infected with Francisella, including induction of alternative activation. Therefore, we next sought to determine if lethal infection with SchuS4 or LVS resulted in induction of alternative activation of macrophages in vivo. Alternative activation of macrophages in the lung is associated with an influx of IL-5 producing eosinophils, and increased expression of Arg1, FIZZ-1 and Ym-1 [21], [22]. Repeated exposure of mice to schistosomal egg antigen (SEA) is an established method to provoke AAMs in vivo [23]–[25]. Therefore, we utilized mice exposed to SEA as positive controls of induction of AAMs for in vivo experiments. As expected, SEA treated mice displayed defined, pulmonary perivasculitis characterized by diffuse perivascular infiltrates of small to moderate numbers of eosinophils (Figure 2A and Table 1). SEA treated mice also had significantly elevated concentrations of IL-5 in the BAL fluid compared to mock treated controls (Figure 2C). Finally, SEA treated animals also had significantly increased expression of Arg1, FIZZ-1 and Ym-1 in the lungs compared to uninfected controls (Figure 2D).

**Table 1:** Pulmonary pathology scores for mice treated with SEA or infected with SchuS4 or LVS

Treatment	SEA	LVS^a^	SchuS4^b^
**Eosinophilic and histiocytic perivasculitis**	**12** c	**0**	**0**

**Day 6 after infection.** ^a^

b **Day 4 after infection.**

c **scores represent combined value of severity of lesion and distribution throughout the lung.**

Mice infected with LVS or SchuS4 had similar numbers of bacteria in their lungs at the time point after infection in which mice required euthanasia, e.g. day 4 for SchuS4 and day 6 for LVS (Figure 2B). However, despite presence of detectable inflammation, neither LVS nor SchuS4 infection resulted in recruitment of eosinophils throughout the course of infection (Figure 2A, Table 1, and data not shown). Further, neither LVS nor SchuS4 infection induced production of IL-5 in the airways at any time after infection (Figure 2C and data not shown). Finally, neither mice infected with LVS nor SchuS4 had significant changes in expression of Ym-1 in the lung at any time point after infection (Figure 2D). Consistent with our in vitro observations, LVS also induced significantly increased expression of Arg1 in mouse lungs compared to mock infected controls (Figure 2D). In addition to Arg1, FIZZ-1 was significantly increased in the lungs of LVS infected mice compared to mock infected controls at each time point after infection (Figure 2D). There was no evidence of significantly increased expression of FIZZ-1 or Arg1 in the lungs of SchuS4 infected mice at any time point after infection (Figure 2D). Together these data demonstrate that neither LVS nor SchuS4 induce alternative activation of macrophages in vivo as defined by (i) presence of eosinophils; (ii) production of IL-5; and (iii) expression of Ym-1, FIZZ-1 and Arg1.

### Arg1 does not contribute to replication of Francisella

**Figure 3:**
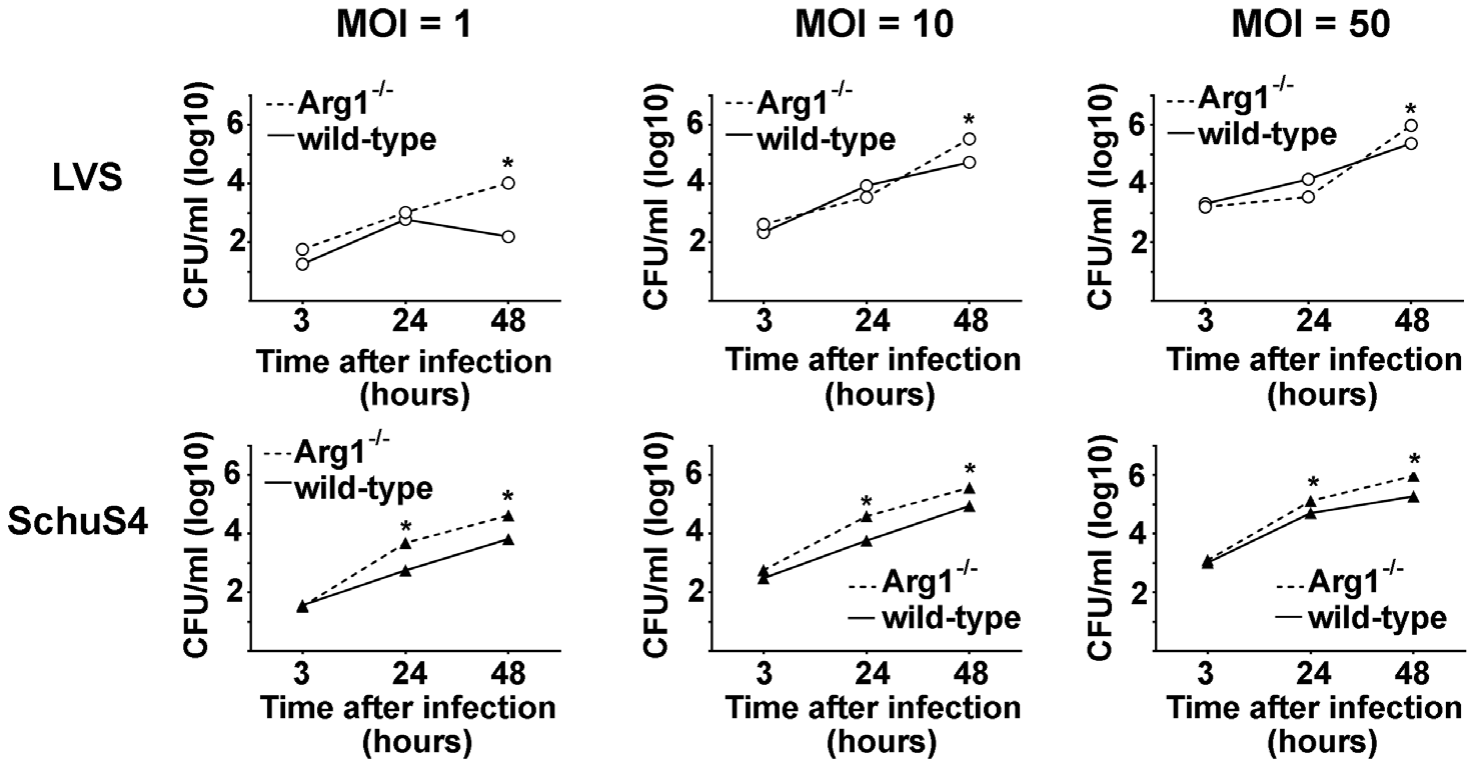
Arg1 does not enhance intracellular replication of Francisella. Primary BMM obtained from Arg1^flox/flox^ (WT) or Arg1^flox/flox^-Tie2Cre (Arg1^-/-^) mice were infected with the indicated MOI of LVS or SchuS4 and intracellular replication was monitored over time. Each data point represents the mean of triplicate samples. *  =  significantly different from WT controls (p<0.05). Error bars represent SEM. Data is representative of three experiments of similar design.

Although we did not observe induction of alternative activation in macrophages infected with LVS or SchuS4, LVS did consistently induce increased expression of Arg1 among infected macrophages. Increased expression of Arg1 independent of alternative activation has been shown to be an important component of pathogenesis for a variety of other intracellular pathogens [26]. Thus, we next examined if Arg1 was important for facilitating intracellular replication of LVS and SchuS4. Macrophages were obtained from mice with targeted deletion of Arg1 in hematopoietic cells in the bone marrow from which macrophages were derived as previously described [26]. Arg1^-/-^ and Arg1^flox/flox^ (WT) macrophages were infected with LVS or SchuS4 at the indicated MOIs and replication of bacteria was assessed over time. Despite induction of Arg1 by LVS in wild type macrophages, absence of Arg1 did not inhibit intracellular replication of LVS or SchuS4 (Figure 3). In fact, we observed a consistent, modest increase in intracellular bacteria 48 hours after infection among Arg1^-/-^ infected cells compared to controls (Figure 3). Thus, Arg1 does not significantly enhance intracellular replication of either LVS or SchuS4.

## Discussion

Appropriate activation of macrophages is an important aspect of host defense against intracellular pathogens. Classically activated macrophages are evoked by Th1 associated cytokines, e.g. IFN-γ, and are characterized by production of nitric oxide and secretion of IL-12, IL-8 and CXCL10 [12]. These classically activated macrophages are associated with control of replication of intracellular bacteria [12]. AAMs are induced by IL-4, IL-13 and IL-5 and characterized by expression of the genes Arg1, Ym-1 and FIZZ-1. AAMs are associated with Th2 immune responses and are thought to contribute to protection against parasitic disease [12]. Due to the requirement of Th1 type immune responses for protection against infection with *F. tularensis* and the ability of AAMs to dampen these responses, it has been suggested that direct induction of AAMs may be a strategy used by *F. tularensis* to limit protective Th1 type immune responses [11].

We comprehensively explored the possibility that both virulent (SchuS4) and attenuated (LVS) strains of Francisella may be able provoke alternative activation of primary cells in vitro and in vivo. We first determined if either SchuS4 or LVS induced alternative activation following infection of homogenous bone marrow derived macrophages in vitro. We did not observe production of cytokines associated with alternative activation, i.e. IL-4 or IL-13 (Figure 1B), nor did we detect significant changes in expression of two of the three genes typically upregulated in AAMs, Ym-1 and FIZZ-1 (Figure 1C). We did observe consistent secretion of pro- and anti-inflammatory cytokines, IL-6, IL-12p40, and IL-10 among BMDM infected with attenuated LVS. Similarly, we also observed increased expression of Arg1. Our in vitro results were recapitulated following in vivo infection with LVS. Mice infected with a lethal dose of LVS had significantly elevated concentrations of IL-6 and IL-12 in their airways and increased expression of Arg1 and FIZZ-1 in the lung. However, we did not detect IL-4, IL-5, or IL-13 at concentrations different from negative controls, nor did we observe changes in expression of Ym-1. Thus, induction of Arg1 by LVS infection was independent of triggering alternative activation in the host.

These results are consistent with recent observations in which infection of peritoneal macrophages with LVS induced a gene expression pattern more closely associated with classically activated macrophages, e.g. upregulation of iNOS, Slc7a2, Arg1 and Ass1, rather than AAMs [27]. However, our results are in disagreement with a previously published report demonstrating induction of IL-4, Arg1, Ym-1 and FIZZ-1 among macrophages infected with LVS [11]. One explanation for this discrepancy is that the earlier report utilized thioglycollate elicited peritoneal macrophages whereas we performed our experiments with primary resting bone marrow derived macrophages. We have observed both increased levels of Arg1 expression among elicited peritoneal macrophages compared to resting peritoneal macrophages as well as an enrichment of contaminating mast cells in preparation of thioglycollate elicited macrophages (CM Bosio and AJ Griffin, unpublished observations). LVS is capable of provoking secretion of IL-4 from mast cells [28]. Thus, it is possible that rather than LVS inducing IL-4 directly from peritoneal macrophages this cytokine was derived from mast cells also present in cultures of peritoneal cells responding to LVS.

Our data do not support a role for alternative activation of macrophages in mediating the pathogenesis of Francisella infection. One explanation for the absence of a role for this macrophage program is that Francisella has multiple strategies for evading the host immune response [29], [30], and that it does not require the use of an AAM program to increase its virulence. For example, by targeting cells at the site of infection that lack CD14, SchuS4 effectively evades initial detection by host [8]. SchuS4 also freely replicates in the cytosol of the host cell without activating host cytoplasmic detection machinery (Bauler TJ and Bosio CM, unpublished data). Finally, SchuS4 directly inhibits activation of specific signal transduction proteins to limit transcription of genes encoding pro-inflammatory cytokines [16], [31]. Together this suggests that provocation of an alternative activation program by the bacterium is not necessary for successful establishment of an anti-inflammatory setting in the host.

Despite our observations that SchuS4 did not induce AAMs, initiation of this program could be an attractive strategy for a pathogen to further advance infection. For example, recent reports have demonstrated that pre-existence of allergic lung inflammation, which largely involves AAMs, significantly enhanced infection mediated by *Streptococcus pneumoniae* compared to non-sensitized mice [32]. In that study, continued potentiation of the AAM phenotype by *S. pneumoniae* infection was thought to contribute to the exacerbation of infection. In agreement with these previous publications, preliminary data from our laboratory suggests that using an exogenous antigen to provoke pulmonary AAM, establishment of AAM populations in the lung prior to SchuS4 infection modestly enhanced pathogenicity of this organism (Griffin AJ and Bosio CM, unpublished data). However, consistent with our findings presented herein, SchuS4 did not further augment the presence of AAMs nor did the bacterium aid in sustaining AAM phenotype over time.

In contrast to the lack of phenotypic and genotypic markers of AAM, we did observe a consistent difference in the ability of attenuated LVS to induce production of various inflammatory cytokines and expression of Arg1 compared to virulent SchuS4. The ability of LVS to induce high concentrations of pro- and anti-inflammatory cytokines was initially surprising since previous data from our laboratory showed an absence of cytokine production among LVS infected mouse macrophages [20], [33]. However, the isolate of LVS used in the current study (ATCC 29684) was utilized to attempt to replicate previous published findings and was different from those used in our prior reports [11], [20], [33], [34]. When compared side-by-side LVS ATCC 29684 provokes greater secretion of cytokines and is more highly attenuated in vivo compared to other isolates of LVS we have used (data not shown). This suggests that the ability of the bacterium to be detected by the host and to induce pro-inflammatory cytokines and Arg1 is inversely correlated to the relative virulence of bacterium, e.g. the more virulent the organism the fewer host pathways it activates. Never-the-less, induction of Arg1 independent of alternative activation has been shown to contribute to the pathogenesis of various intracellular pathogens [26], [27]. Thus, despite the marked attenuation of LVS ATCC 29684 we addressed the possibility that induction of Arg1 by this bacterium may enhance its intracellular replication. Unlike other intracellular organisms, we did not observe a role in Arg1 promoting intracellular replication of either SchuS4 or LVS (Figure 3). Instead, we observed a modest, but consistent, increase in numbers of intracellular bacteria among macrophages deficient for Arg1. This suggested that Arg1 may not be beneficial in exacerbating Francisella infection. In conclusion, our data point toward an absence of a role for alternative activation and Arg1 in promoting Francisella infection and suggest that evasion and suppression of host responses in general is a central mechanism of virulence mediated by this bacterium.
